# Late Nontraumatic Dissociation of the Femoral Head and Trunnion in a Total Hip Arthroplasty

**DOI:** 10.1155/2015/738671

**Published:** 2015-05-11

**Authors:** Simon J. M. Parker, Wasim Khan, Simon Mellor

**Affiliations:** ^1^Oxford University Hospitals NHS Trust, John Radcliffe Hospital, Headley Way, Oxford OX3 9DU, UK; ^2^Royal Free London NHS Foundation Trust, Barnet Hospital, Wellhouse Lane, Barnet, Hertfordshire EN5 3DJ, UK

## Abstract

*Background*. Modular total hip arthroplasties are increasingly popular because customisation allows optimal restoration of patient biomechanics. However, the introduction of component interfaces provides greater opportunities for failure. We present a case of late nontraumatic dissociation of the head-neck interface, more than 10 years after insertion. *Case Description*. A 58-year-old woman had a left metal-on-metal total hip arthroplasty in 2002 for hip dysplasia. Following an uneventful 10-year period, she presented to hospital in severe pain after standing from a seated position, and radiographs demonstrated complete dissociation of the modular femoral head from the stem, with the femoral head remaining in its cup. There was no prior trauma or infection. Mild wear and metallosis were present on the articulating surface between the femoral head and trunnion. Soft tissues were unaffected. *Discussion and Conclusions*. This is the latest occurrence reported to date for nontraumatic component failure in such an implant by more than 7 years. The majority of cases occur in the context of dislocation and attempted closed reduction. We analyse and discuss possible mechanisms for failure, aiming to raise awareness of this potential complication and encouraging utmost care in component handling and insertion, as well as the long term follow-up of such patients.

## 1. Introduction

Modular total hip arthroplasties (THA) incorporating a single head-neck taper design have been used for decades to allow for adjustment of leg length and offset and facilitate revision of an articulation whilst retaining a well-fixed stem.

The advantages of femoral head modularity are considered to outweigh the reported failures, a position reflected in the more recent progression to double-taper designs, which add an additional neck-stem taper junction to allow for further customisation of femoral anteversion and optimal restoration of soft tissue tension and patient biomechanics [1]. However, mechanisms of failure of modular hip designs are well described, the most common being crevice corrosion and/or fretting corrosion, particularly in devices with a titanium alloy stem and cobalt-chromium femoral head [2–5]. Other failings include complete dissociation of the head and neck interface, which has previously been reported, usually in the context of dislocation and attempts at closed reduction [6–10]. One previous case report describes a nontraumatic dissociation at 3 years whilst rising from a seated position (52 mm polyethylene acetabular cup and 32 mm modular head; cemented Protasul S-30 stem, PROTEK) [11].

We report a late case of nontraumatic dissociation of the femoral head-neck taper, more than 10 years after insertion.

## 2. Case Report

**Figure 1:**
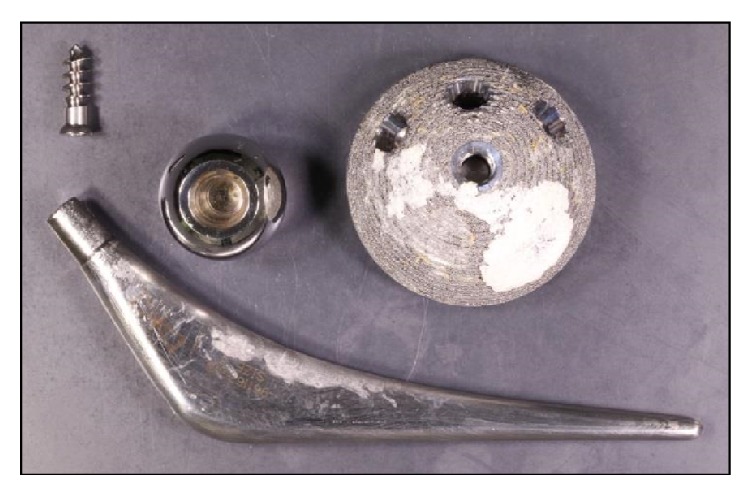
Retrieved components from the original total hip arthroplasty.

A 58-year-old woman with a BMI of 35 kg/m^2^ suffered with hip dysplasia and had a left total hip replacement in 2002 at another institution. DePuy International Ultima components were used including a cemented polished tapered C-Stem femoral component with a 28 mm diameter cobalt-chrome (CoCr) modular head, articulating with a 28 mm CoCr acetabular bearing surface secured in a 54 mm titanium alloy uncemented shell (Figure 1). She had remained well since her operation and had been coping with her new hip without significant difficulty. Her Oxford Hip score immediately before the event was 35/48. She reported occasional mild pain and limp and some mild to moderate difficulty with everyday activities such as shopping, housework, and most notably putting on shoes and socks. Her symptoms, however, did not limit her activities of daily living and had therefore not been followed up or further investigated at her original institution.

**Figure 2:**
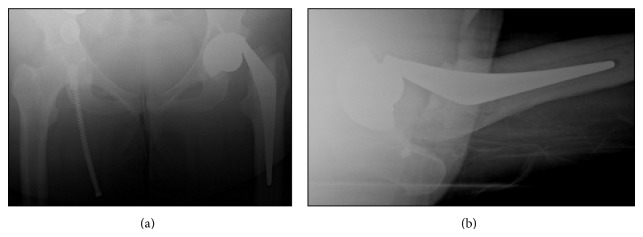
(a) AP and (b) lateral radiographs of the dissociated THA.

In October 2013, more than 10 years after her initial operation, she stood from a seated position and heard a loud “clunk,” followed by pain in the hip associated with difficulty mobilising. Radiographs demonstrated a complete dissociation of the modular femoral head from the stem, which was dislocated (Figure 2). The femoral head remained in its cup. There was no evidence of osteolysis or loosening of the cup or stem. She was systemically well with serum inflammatory markers only marginally elevated (CRP 11 mg/L; normal value <10 mg/L).

**Figure 3:**
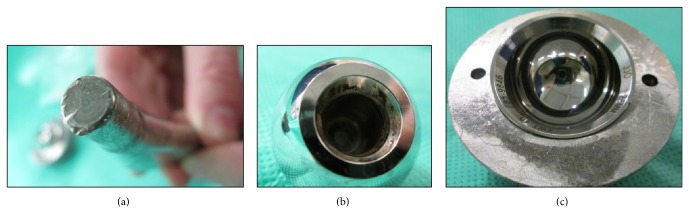
(a) Visible wear and damage on the trunnion (the significant deformation at the tip was caused by surgical removal of the well-fixed stem), (b) black debris in the articulating surface of the femoral head, and (c) minimal wear or damage to the metal liner.

We performed a revision left total hip replacement via a posterior approach, using the new model DePuy C-Stem AMT, 36 mm head and Pinnacle 60 mm shell with polyethylene liner. We noted mild wear on the trunnion, prompting concomitant stem revision, and black debris around the inner surface of the head, likely to represent mild metallosis (Figure 3). The cup and C-Stem were both well fixed. There was no evidence of wear or impingement affecting the outer articulating surface of the head or metal liner. The soft tissues were grossly unaffected.

**Figure 4:**
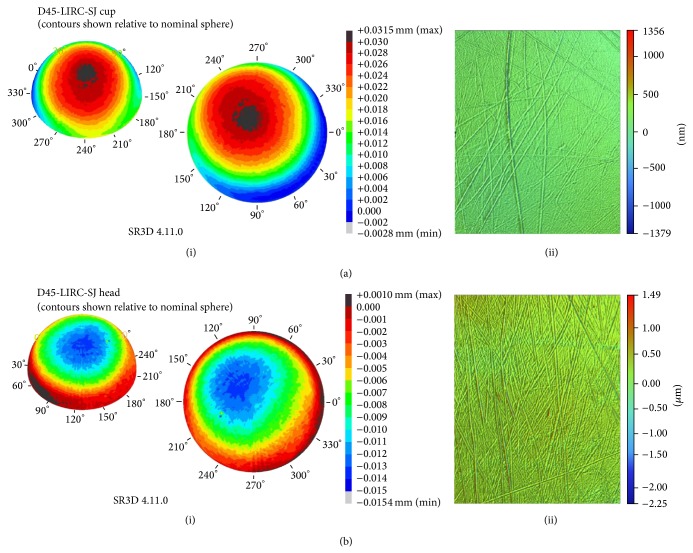
Graphical representation of (a)(i) cup wear profile, (a)(ii) cup surface roughness, (b)(i) femoral head wear profile, and (b)(ii) femoral head surface roughness from the LIRC analysis.

The removed components were sent to the London Implant Retrieval Centre (LIRC) for metrological analysis using coordinate-measuring machine (CMM) dimensional measurements and optical interferometry roughness measurements (reference D45-LIRC0SJ) (Figure 4). Acetabular cup analysis demonstrated roughness measurements of up to 0.023 *μ*mRa. Femoral head analysis demonstrated roughness measurements of up to 0.082 *μ*mRa. Unfortunately the methods utilised at this centre prohibit analysis of the head-taper junction.

## 3. Discussion

The use of modular components provides greater flexibility for optimisation of patient biomechanics when performing a THA, but the introduction of additional interfaces also adds further opportunities for failure. Dislocation remains the most common complication, with highlighted risk factors including surgical approach, restoration of tissue tension, prosthetic design, and orientation of components [7, 9]. Unique to modular designs, however, is the risk of dissociation, which appears most frequently in association with dislocation and attempts at closed reduction, and can occur at the fixed acetabular shell-liner interface [9, 12–17], the femoral head-neck interface [7, 9, 10], or the femoral neck-stem interface [18].

In head-neck dissociation, postulated mechanisms include strong distraction forces caused by the inferior edge of the femoral head becoming caught on the rim of the acetabular component during reduction of a dislocation [19] and excessive external rotation in the presence of a constrained liner, in which the elevated liner would act as a fulcrum at the head and neck junction [6]. However, we know that a considerable force is required to achieve dissociation of a well impacted head, with cited values including 3003 ± 623 N [20] and 427 kg [21]. Adequate impaction of the head-neck interface is achieved by a strong blow with a 0.5 kg mallet and a femoral head driver, in combination with the cyclic impaction loading in the normal activities of daily living. Spinnickie and Goodman, who reported a case of head-neck dissociation in a patient with poliomyelitis, attributed the complication to a lack of cyclic compression and therefore inadequate head-neck impaction, secondary to their patient's limited mobility [22]. Another theory is that “pumping phenomenon,” a mechanical engineering term, may be responsible for rising air pressure in the inner head, giving rise to an unlocked tapered neck where there is air trapping between the inner head and tapered stem. Smaller forces are required for dissociation as sealed air pushes back on the neck and unlocks the taper mechanism of the inner head, causing separation [21]. However, in the case presented here, we see the complication arise in a nontraumatic scenario, after 10 years of full weight bearing, which makes the possibilities of high distraction forces and a pumping phenomenon seem unlikely.

The only other case of late modular head-neck dissociation in THA known to the authors occurred at 3 years postoperatively [11]. Karaismailoglu et al. noted moderate, Booker's class III, ectopic bone formation in the abductor area and attribute the nontraumatic dissociation to the heterotopic ossification of the hip producing greater stress on the femoral head, resulting in easier detachment. We noted no such ossification in the case described here.

Corrosion at the head-neck interface has been described since the 1980s [23], and numerous reports of corrosion have been documented in retrieval studies, with metallic debris from modular connections being primarily implicated both in component corrosion and surrounding soft tissue damage [24]. More recently, Donaldson et al. have suggested that assembly conditions, patient activity, and design factors may also play a role, identifying that fretting corrosion is significantly affected by small changes in angular mismatch, centre offset, and patient body weight [25]. It is likely that both design and patient factors contributed to the outcome presented in this case. Notable damage and loss of substance from the trunnion are visible, as well as black marks of metallosis on the inner articulating surface of the femoral head. These changes may have compromised the taper lock, which, coupled with surrounding soft tissue weakness, may have allowed the head to disengage from the stem. It is possible that there was also a degree of impingement associated with having a 28 mm head in a 54 mm cup. Indeed, this would fit with the patient prior history of having difficulties in hip flexion (such as when putting on shoes and socks, e.g.,) and the incident occurring when rising from a seated position.

There were few prior symptoms to suggest an impending complication. Mann et al. recently described two cases of severe metal-induced osteolysis and surrounding soft tissue damage secondary to trunnion wear at 10 and 13 years after unipolar endoprosthesis insertion [26], one of which presented acutely with dissociation and no prior symptoms and one with leg weakness and limping but no dissociation. In our case, the only potential prior signs of complication were mild pain and limp and moderate difficulty with certain activities of daily living.

The main limitation of our presentation is our lack of facility for measuring metal ion levels, which may have provided further prior clues as to the degree of wear occurring, as recent studies have shown increased levels of trunnion corrosion associated with increased serum metal ion levels [27, 28].

This paper reports a case of head-neck dissociation in a THA more than 10 years after initial operation, which, to the author's knowledge, is the latest occurrence reported to date for this type of prosthesis by a difference of more than 7 years. Furthermore, there were minimal warning signs of this impending complication. We recommend the utmost care when handling and inserting the components during surgery to avoid potential inadvertent damage to the bearing surfaces and trunnion, as well as careful component selection and intraoperative testing to minimise impingement. With ongoing research in wear mechanisms and components design we will likely see reductions in this type of complication future, but we would encourage the long term follow-up of these patients, maintaining a high index of suspicion for signs of wear and potentially imminent complications.

## References

[1] Dunbar M. J. (2010). The proximal modular neck in THA: a bridge too far: affirms. *Orthopedics*.

[2] Goldberg J. R., Gilbert J. L., Jacobs J. J., Bauer T. W., Paprosky W., Leurgans S. (2002). A multicenter retrieval study of the taper interfaces of modular hip prostheses. *Clinical Orthopaedics and Related Research*.

[3] Salvati E. A., Lieberman J. R., Huk O. L., Evans B. G. (1995). Complications of femoral and acetabular modularity. *Clinical Orthopaedics and Related Research*.

[4] Cook S. D., Barrack R. L., Clemow A. J. T. (1994). Corrosion and wear at the modular interface of uncemented femoral stems. *The Journal of Bone and Joint Surgery—British Volume*.

[5] Collier J. P., Mayor M. B., Williams I. R., Surprenant V. A., Surprenant H. P., Currier B. H. (1995). The tradeoffs associated with modular hip prostheses. *Clinical Orthopaedics and Related Research*.

[6] Namba R. S., van der Reis W. L. (2000). Femoral head and neck dissociation after a total hip arthroplasty with a constrained acetabular liner. *Orthopedics*.

[7] Woolson S. T., Pottorff G. T. (1990). Disassembly of a modular femoral prosthesis after dislocation of the femoral component. A case report. *The Journal of Bone and Joint Surgery—American Volume*.

[8] Pellicci P. M., Haas S. B. (1990). Disassembly of a modular femoral component during closed reduction of the dislocated femoral component. A case report. *The Journal of Bone and Joint Surgery—American Volume*.

[9] Star M. J., Colwell C. W., Donaldson W. F., Walker R. H. (1992). Dissociation of modular hip arthroplasty components after dislocation. A report of three cases at differing dissociation levels. *Clinical Orthopaedics and Related Research*.

[10] Chu C.-M., Wang S.-J., Lin L.-C. (2001). Dissociation of modular total hip arthroplasty at the femoral head-neck interface after loosening of the acetabular shell following hip dislocation. *Journal of Arthroplasty*.

[11] Karaismailoglu T. N., Tomak Y., Gulman B. (2001). Late detachment modular femoral component after primary total hip replacement. *Archives of Orthopaedic and Trauma Surgery*.

[12] Cameron H. U. (1993). Dissociation of a polyethylene liner from an acetabular cup. *Orthopaedic Review*.

[13] Wilson A. J., Monsees B., Blair V. P. (1988). Acetabular cup dislocation: a new complication of total joint arthroplasty. *The American Journal of Roentgenology*.

[14] Ries M. D., Collis D. K., Lynch F. (1992). Separation of the polyethylene liner from acetabular cup metal backing: a report of three cases. *Clinical Orthopaedics and Related Research*.

[15] Bueche M. J., Herzenberg J. E., Stubbs B. T. (1989). Dissociation of a metal-backed polyethylene acetabular component. A case report. *Journal of Arthroplasty*.

[16] Ferenz C. C. (1988). Polyethylene insert dislocation in a screw-in acetabular cup. A case report. *Journal of Arthroplasty*.

[17] O'Brien R. F., Chess D. (1992). Late dissambly of a modular acetabular component. A case report. *Journal of Arthroplasty*.

[18] Kouzelis A., Georgiou C. S., Megas P. (2012). Dissociation of modular total hip arthroplasty at the neck-stem interface without dislocation. *Journal of Orthopaedics and Traumatology*.

[19] Ito H., Minami A., Kondo E., Fujita M., Ubayama Y., Matsuno T. (2001). Destruction of acetabular bone caused by early failure of a constrained acetabular component. *Journal of Arthroplasty*.

[20] Lieberman J. R., Rimnac C. M., Garvin K. L., Klein R. W., Salvati E. A. (1994). An analysis of the head-neck taper interface in retrieved hip prostheses. *Clinical Orthopaedics and Related Research*.

[21] Shiga T., Mori M., Hayashida T., Fujiwara Y., Ogura T. (2010). Disassembly of a modular femoral component after femoral head prosthetic replacement. *Journal of Arthroplasty*.

[22] Spinnickie A., Goodman S. B. (2007). Dissociation of the femoral head and trunion after constrained conversion total hip arthroplasty for poliomyelitis. *Journal of Arthroplasty*.

[23] Lucas L. C., Buchanan R. A., Lemons J. E. (1981). Investigations on the galvanic corrosion of multialloy total hip prostheses. *Journal of Biomedical Materials Research*.

[24] Cooper H. J., Della Valle C. J., Berger R. A. (2012). Corrosion at the head-neck taper as a cause for adverse local tissue reactions after total hip arthroplasty. *The Journal of Bone and Joint Surgery—American Volume*.

[25] Donaldson F. E., Coburn J. C., Siegel K. L. (2014). Total hip arthroplasty head-neck contact mechanics: a stochastic investigation of key parameters. *Journal of Biomechanics*.

[26] Mann M. A., Tanzer D., Tanzer M. (2013). Severe metal-induced osteolysis many years after unipolar hip endoprosthesis hip. *Clinical Orthopaedics and Related Research*.

[27] Bolland B. J. R. F., Culliford D. J., Langton D. J., Millington J. P. S., Arden N. K., Latham J. M. (2011). High failure rates with a large-diameter hybrid metal-on-metal total hip replacement: clinical, radiological and retrieval analysis. *The Journal of Bone & Joint Surgery—British Volume*.

[28] Garbuz D. S., Tanzer M., Greidanus N. V., Masri B. A., Duncan C. P. (2010). The john charnley award: metal-on-metal hip resurfacing versus large-diameter head metal-on-metal total hip arthroplasty: a randomized clinical trial. *Clinical Orthopaedics and Related Research*.

